# Supplementary material for: Molecular characterization and expression profiling of transformer 2 and fruitless-like homologs in the black tiger shrimp, Penaeus monodon
Source: PeerJ. 2022 Feb 17;10:e12980. doi: 10.7717/peerj.12980 (PMC8858584; doi:10.7717/peerj.12980)
Supplement: Supplemental Information 1 — Nucleotide (above) and deduced amino acid (below) sequences of PmOvtra 2. The nucleotide sequences encoding the start codon are underlined, and the stop codon are underlined with an asterisk (*) below. The RS-1 and RS-2 regions are blue and green underlined, respectively. The RRM domain is marked with gray shadow located at 101-180 aa. [file peerj-10-12980-s001.docx]

1 AGCGAGGAGCGAGTGCGGACGGACGGACTGCGTCTCGGTTTTCCTCTCGTTTGACTGCGATTTTGCCGCGAGATTGAGTCGTTCCGGAGGCGGAGGCGAGGTACAGAAGA

111 ATGAGTCGATCACCGCAACATGCTGTGGCCAATGGAGCCTCACCAGTTCGTGAAAGGTCGAGGGACCCTTCATTTTCCCGCTCAAGATCACGCTCCAGAGACCGACGTGAAACCTACAAA

1 M S R S P Q H A V A N G A S P V R E R S R D P S F S R S R S R S R D R R E T Y K

231 CACTCCTCTAGAAGTTCTGGTTCCCCACGTTACCGCGAGGACCGCTACCGAGAGGACAAATATTCATCATCACGCCGTAGAGATTCTCGTTCTCCATCTTACTCCAGAAATAGAAGGAGT

41 H S S R S S G S P R Y R E D R Y R E D K Y S S S R R R D S R S P S Y S R N R R S

351 AACAGGTCACCAATGTCGAATCGCCGCCGCCATCATGGAAGCAGAGAGGACCCGTCCCCGAGCAACTGCCTTGGCATCTTTGGACTCTCTCTCTACACAACCGAGAGACAGCTTCATCAC

81 N R S P M S N R R R H H G S R E D P S P S N C L G I F G L S L Y T T E R Q L H H

471 CTCTTTGGCAAATATGGTCATATCAACGAAGTTCAAGTTGTACTAGATGCTAAAACTGGTCGATCAAGGGGATTTGCATTTATCTACTTTGATCATGTGGATGATGCCACAGAAGCCAAG

121 L F G K Y G H I N E V Q V V L D A K T G R S R G F A F I Y F D H V D D A T E A K

591 GAGCAGTGTACTGGGATGGAGATTGATGGAAGACGGATCAGAGTAGATTATTCCATCACAGAGAGAGCCCACACACCCACTCCTGGCATATACATGGGTCGACCAACTTATTCTAACAAT

161 E Q C T G M E I D G R R I R V D Y S I T E R A H T P T P G I Y M G R P T Y S N N

711 GGTCGTAGAGGTGGTGGTGGAGGTGGAGGAGGTCGCCACAGAGGTGGATATGGAGGAGGCAGTCGCAGTCGCCGGTCCCCTCCACGCAGATCATACCGCTCACGCTCTCGGTCATACTCT

201 G R R G G G G G G G G R H R G G Y G G G S R S R R S P P R R S Y R S R S R S Y S

831 CCACGTCGCTACAGCCGGTATTGAGGTGCTGGTGTCACTTTAGTTTGATTATAACTAGAAACATGAACTGTATTTAATGTAACTACACATTCTAACAAGATCAAAGCACTCTGATATAAA

241 P R R Y S R Y *

951 AAGCATATTTTATCCTGTTAGAATGCAACATTAGATATCCAGCAGTTTCATGGTTTTCAAAGTTTAGCAGACCTATATTTAAGGACAAGCTGGTTGTTTTGACCAACAGTTTTTCTATGT

1071 ACTGAGGAAGTATTCCTCAAGATATAATGTTCTTTGCATCAGGTATCAAGATATTTTTCGTTAAAAGATGACCAGAAGTGAATATCTGCTGTGTCTTGAGATATAATAAGCATTTTATAT

1191 ATGGAGAGGAATGAAAGAAACAAGATTATTTTCAAATTTAGGACTTTTTCATCTTATCAGGAGAATCCAGAATGAATATTTCTATTTTTTGTCTTCTGTGTATTTCTTTTCTTGTTTTAA

1311 GATGAGTTTTATTAGTATTTTGGAGTGATTCTAGGTCAATAATCACCCTTTCTGTAACACATTATAAAATGATCAATAAAGATGAAACAAAAGGGTTGTTTAATAGGCATTAGTTCTCCT

1431 TGTAGATAGTTGATTTACAATACCTTTGTATGTTGAGAACTTCTTTGATTGTTTCATCCATCTCAGTTTGTTTCGTAAAAGACATTGCTCACGAGTTTGCCACAACTTGGTATATACTGA

1551 TTATGTTCTGTTATGGTTTCCCTTTTTTCTCTTTTTATCTTTTCATAAGTGAATGACAAACATGTGCTTATTCATTGGTAAGTGAAAGTAATGTGAAAGTGATTAAAGGTGACCCATGTT

1671 TAGACTAGATGGAGTTGAGGTGGGACATCCTCATTAAAGGAAAAAGAATTACTTAAGTTGAAGCTTAAGACTGTGTACGTCAGAAGTTTTTTTGTGGTTCGCTC
