# Supplementary material for: Molecular characterization and expression profiling of transformer 2 and fruitless-like homologs in the black tiger shrimp, Penaeus monodon
Source: PeerJ. 2022 Feb 17;10:e12980. doi: 10.7717/peerj.12980 (PMC8858584; doi:10.7717/peerj.12980)
Supplement: Supplemental Information 2 — Nucleotide (above) and deduced amino acid (below) sequences of Pmfru-1 and Pmfru-2. The nucleotide sequences encoding the start codon are underlined and the stop codon are underlined with an asterisk (*). The predicted BTB and ZF domains were marked with gray shadow located at 31-126 aa, 339-389 aa in Pmfru-1, and at 31-126 aa and (373-395, 401-424) aa in Pmfru-2. [file peerj-10-12980-s002.docx]

1 GCTACTTGCCATCGATTGTTTACTTGTCTGTCCTGGAGTGCCCTTTGGAAACCATTTTTAAAAGACAAATTACACTGACGCTTCGAGAAAGACATTAAGAG *Pmfru-1*

1 GATCGATTGTTTACTTGTCTGTCCTGGAGTGCCCTTTGGAAACCATTTTTAAAAGACAAATTACACTGACGCTTCGAGAAAGACATTAAGAG *Pmfru-2*

102 ATGGAGGAGGGTATACTCTCACTGCGTTGGAATAATCATCGATCAACATTTTTCCATATTTTATCTACACTTCATAGGAAGGAACTTTACAGTGATGTAACATTAGCATGCAATGGTAAA *Pmfru-1*

1 M E E G I L S L R W N N H R S T F F H I L S T L H R K E L Y S D V T L A C N G K

93 ATGGAGGAGGGTATACTCTCACTGCGTTGGAATAATCATCGATCAACATTTTTCCATATTTTATCTACACTTCATAGGAAGGAACTTTACAGTGATGTAACATTAGCATGCAATGGTAAA *Pmfru-2*

1 M E E G I L S L R W N N H R S T F F H I L S T L H R K E L Y S D V T L A C N G K

222 TTTTTCCCAGTACACAAGTTGGTGCTTTCAGTGTGTAGTGAATATTTTGAGGAAATGTTTAAGCAGACCACATGTAAACATCCCATAATAGTACTTAAGGATATTCTTCACGATGACCTT *Pmfru-1*

41 F F P V H K L V L S V C S E Y F E E M F K Q T T C K H P I I V L K D I L H D D L

213 TTTTTCCCAGTACACAAGTTGGTGCTTTCAGTGTGTAGTGAATATTTTGAGGAAATGTTTAAGCAGACCACATGTAAACATCCCATAATAGTACTTAAGGATATTCTTCACGATGACCTT *Pmfru-2*

41 F F P V H K L V L S V C S E Y F E E M F K Q T T C K H P I I V L K D I L H D D L

342 GAAGCGCTTCTCAATTACATGTATGCGGGTGAGGCAAATGTAGCGCAAAATGACCTCGCCAGATTAATTAAAGCTGCTGAGTGTTTAAGAATCAAAGGTCTAGCCGTCCCGGATGAAGCC *Pmfru-1*

81 E A L L N Y M Y A G E A N V A Q N D L A R L I K A A E C L R I K G L A V P D E A

333 GAAGCGCTTCTCAATTACATGTATGCGGGTGAGGCAAATGTAGCGCAAAATGACCTCGCCAGATTAATTAAAGCTGCTGAGTGTTTAAGAATCAAAGGTCTAGCCGTCCCGGATGAAGCC *Pmfru-2*

81 E A L L N Y M Y A G E A N V A Q N D L A R L I K A A E C L R I K G L A V P D E A

462 CCCCCTTCCAGTGAGAGTAAAAGATCCCATACTGAGGGGCTGAGAGAGGAAGCGCCTCACCCCAAGCGGCGGAAACATGATGACAGCTCATCAGCCTCCTCCAAGTCGAGCCAAGGGCGA *Pmfru-1*

121 P P S S E S K R S H T E G L R E E A P H P K R R K H D D S S S A S S K S S Q G R

453 CCCCCTTCCAGTGAGAGTAAAAGATCCCATACTGAGGGGCTGAGAGAGGAAGCGCCTCACCCCAAGCGGCGGAAACATGATGACAGCTCATCAGCCTCCTCCAAGTCGAGCCAAGGGCGA *Pmfru-2*

121 P P S S E S K R S H T E G L R E E A P H P K R R K H D D S S S A S S K S S Q G R

582 CAGTCAGAGGATGAACCCAAAGATAAAAATTGCAAGGAATTGCCTAGCTGCATGGAACAACAGCAACAACATAGTGGAAGATATAGTGGGCAAAGTACAGGATTACAACAAATAACGAGT *Pmfru-1*

161 Q S E D E P K D K N C K E L P S C M E Q Q Q Q H S G R Y S G Q S T G L Q Q I T S

573 CAGTCAGAGGATGAACCCAAAGATAAAAATTGCAAGGAATTGCCTAGCTGCATGGAACAACAGCAACAACATAGTGGAAGATATAGTGGGCAAAGTACAGGATTACAACAAATAACGAGT *Pmfru-2*

161 Q S E D E P K D K N C K E L P S C M E Q Q Q Q H S G R Y S G Q S T G L Q Q I T S

702 CCTGAACTACAACTTGAATTAGAAATGGGGAGATCAAGTCAAGACGACAACAGTGCTACTCAAGATCTTGCTGAGGTTGTCCTGGATGAACAGCCGCTGATCAAAGAGGAGATCCAGGAA *Pmfru-1*

201 P E L Q L E L E M G R S S Q D D N S A T Q D L A E V V L D E Q P L I K E E I Q E

693 CCTGAACTACAACTTGAATTAGAAATGGGGAGATCAAGTCAAGACGACAACAGTGCTACTCAAGATCTTGCTGAGGTTGTCCTGGATGAACAGCCGCTGATCAAAGAGGAGATCCAGGAA *Pmfru-2*

201 P E L Q L E L E M G R S S Q D D N S A T Q D L A E V V L D E Q P L I K E E I Q E

822 CCCAAACATGAACATGATGACGACATAACGCACCAAACGGACTCGGAGGCAAGCATCAGTTTCGACCCCCTCAATTCTGGGGATGAAAGAGGAGGGGGTACAGGCATATATGACCCACAG *Pmfru-1*

241 P K H E H D D D I T H Q T D S E A S I S F D P L N S G D E R G G G T G I Y D P Q

813 CCCAAACATGAACATGATGACGACATAACGCACCAAACGGACTCGGAGGCAAGCATCAGTTTCGACCCCCTCAATTCTGGGGATGAAAGAGGAGGGGGTACAGGCATATATGACCCACAG *Pmfru-2*

241 P K H E H D D D I T H Q T D S E A S I S F D P L N S G D E R G G G T G I Y D P Q

942 CTGATGGTCTCACACCCACAGAGTGTCCTACAAGATATCATGGTGCAAGGGGTCCCGGGCCCTTCTGGACTGCCCACGGACTCCATCACCAGCTGGGATTCAGGTGGAAATGTAGGCTTT *Pmfru-1*

281 L M V S H P Q S V L Q D I M V Q G V P G P S G L P T D S I T S W D S G G N V G F

933 CTGATGGTCTCACACCCACAGAGTGTCCTACAAGATATCATGGTGCAAGGGGTCCCGGGCCCTTCTGGACTGCCCACGGACTCCATCACCAGCTGGGATTCAGGTGGAAATGTAGGCTTT *Pmfru-2*

281 L M V S H P Q S V L Q D I M V Q G V P G P S G L P T D S I T S W D S G G N V G F

1062 TCGCTAGAAGGATTCACAGGCGAGGATGCGAGGACGACCCAGGCCATGAGGGGACCCAAACGATCTCTTGCTTGGGACCACTTTGCGGAAGTCCAACTTAATGGCAGAACAGTAAAGGTC *Pmfru-1*

321 S L E G F T G E D A R T T Q A M R G P K R S L A W D H F A E V Q L N G R T V K V

1053 TCGCTAGAAGGATTCACAGGCGAGGATGCGAGGACGACCCAGGCCATGGATGTGCGAGGCAGTTATAGGCGAAGCCAGTTTGGAAGTAGGTGTGGCAGTGGAGCGACGGGTGGCATGGCT *Pmfru-2*

321 S L E G F T G E D A R T T Q A M D V R G S Y R R S Q F G S R C G S G A T G G M A

1182 CAATGCAAGCACTGTGAAAGGTACCTCTCCTTCAACAGAAACACTTCAGGAATGGTCAGACATCTCGATACAGTGCACAACATCACCATTAATGTCAAATGAAACATGGGAAAGTTTAGG *Pmfru-1*

361 Q C K H C E R Y L S F N R N T S G M V R H L D T V H N I T I N V K *

1173 GGCGGCACCAGAGGGGCACAAGTTGAAGGTGGAACCCACCCATGTGTGCTGTGCCAGAAGCGCTTCACCAGCCGCCAGGACCTGAGACGCCACGTCCGCACTCACACAGGAGAGAGGCCA *Pmfru-2*

361 G G T R G A Q V E G G T H P C V L C Q K R F T S R Q D L R R H V R T H T G E R P

1302 CAGGA *Pmfru-1*

1293 TACCAGTGTCCGCTTTGCCCTCACCGTGCGGCGCTCAAGGGCAACATCAAGAAGCACATCATAGCAGTCCACAGAGACATAACCCCGACTGCAGCTGAGGCTGCAGCAGTTCTTGAGAAA *Pmfru-2*

401 Y Q C P L C P H R A A L K G N I K K H I I A V H R D I T P T A A E A A A V L E K

1413 GAATCATTCCAGGCAGCTGACAACAATGCTGGTGCTGCTACCACCTCCAACACTGGTGTTGGTGTTGAGCTCTTACAGAACTGTTCTTACTCACCAGAGAGTTTCCCTAATTGTTGAAAG *Pmfru-2*

441 E S F Q A A D N N A G A A T T S N T G V G V E L L Q N C S Y S P E S F P N C *

1533 ATATGTGGAAGCTTTGTAGAAGAATTTTTTTTGACAGAGAGATATTAAGTAATTTCCATAATTGGTTATAATCCTCATTACCATCTTGAATATTATTTATGAATTGATATATGAGACATT *Pmfru-2*

1653 ATTTTTTTTAAGTGATATATGTAATTATCCTTACTGGTAGGTCTTAGCTGTATCTATGATATATTGATTTAAGTTACAGTGTAAAAAAATACCAGGCACACCTCATAACCCAAAAGTAAA *Pmfru-2*

1773 TGAAAATGCATTAGGGATATGATATACAAGGTTTGCCTGTATAACAGTTCAGTACAAGCACTATTACACAAGTAAACTTCCTGAAG *Pmfru-2*
