# Supplementary figures and images for: Molecular characterization and expression profiling of transformer 2 and fruitless-like homologs in the black tiger shrimp, Penaeus monodon
Source: PeerJ. 2022 Feb 17;10:e12980. doi: 10.7717/peerj.12980 (PMC8858584; doi:10.7717/peerj.12980)

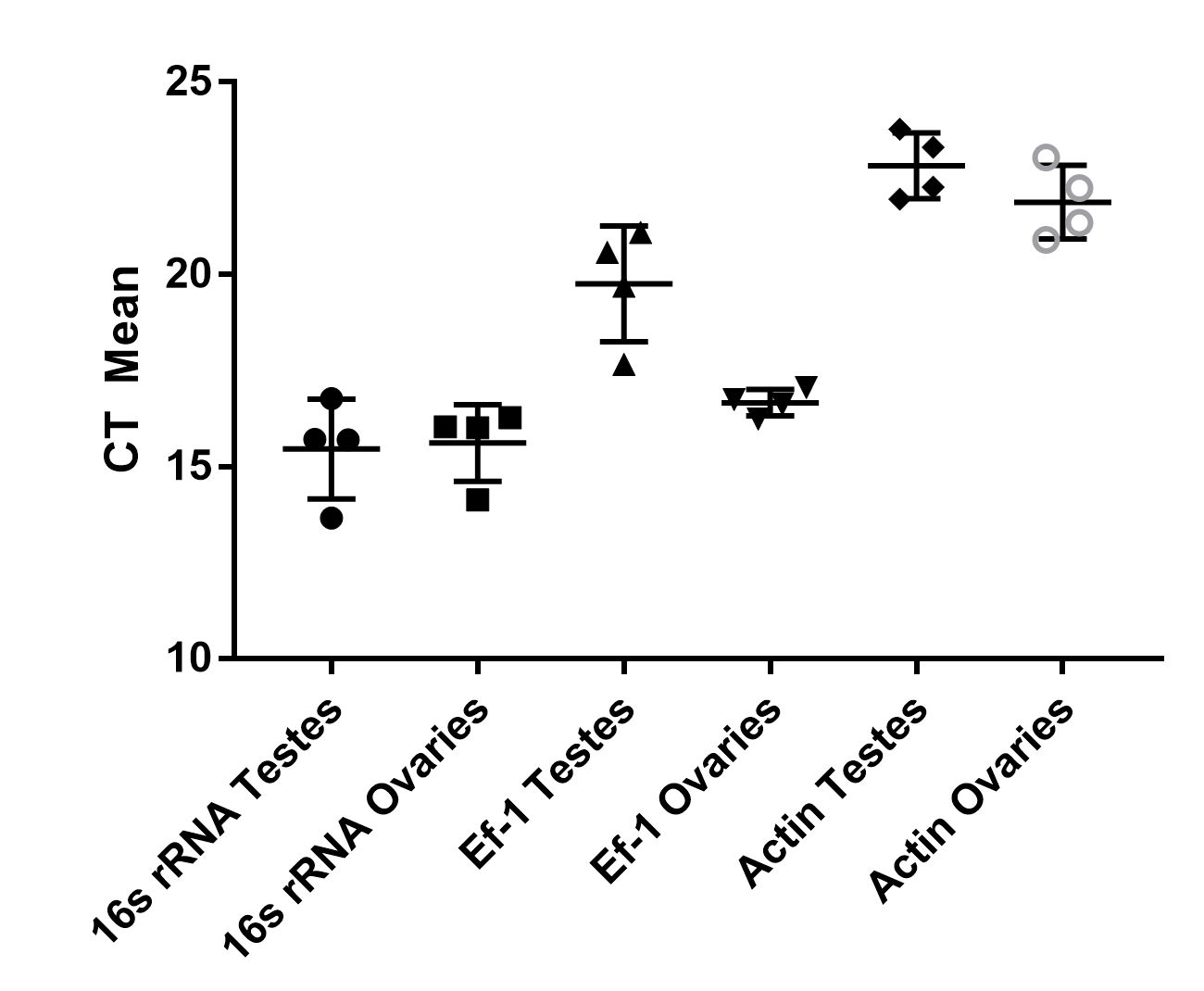

Supplement: Supplemental Information 3 [file peerj-10-12980-s003.png]

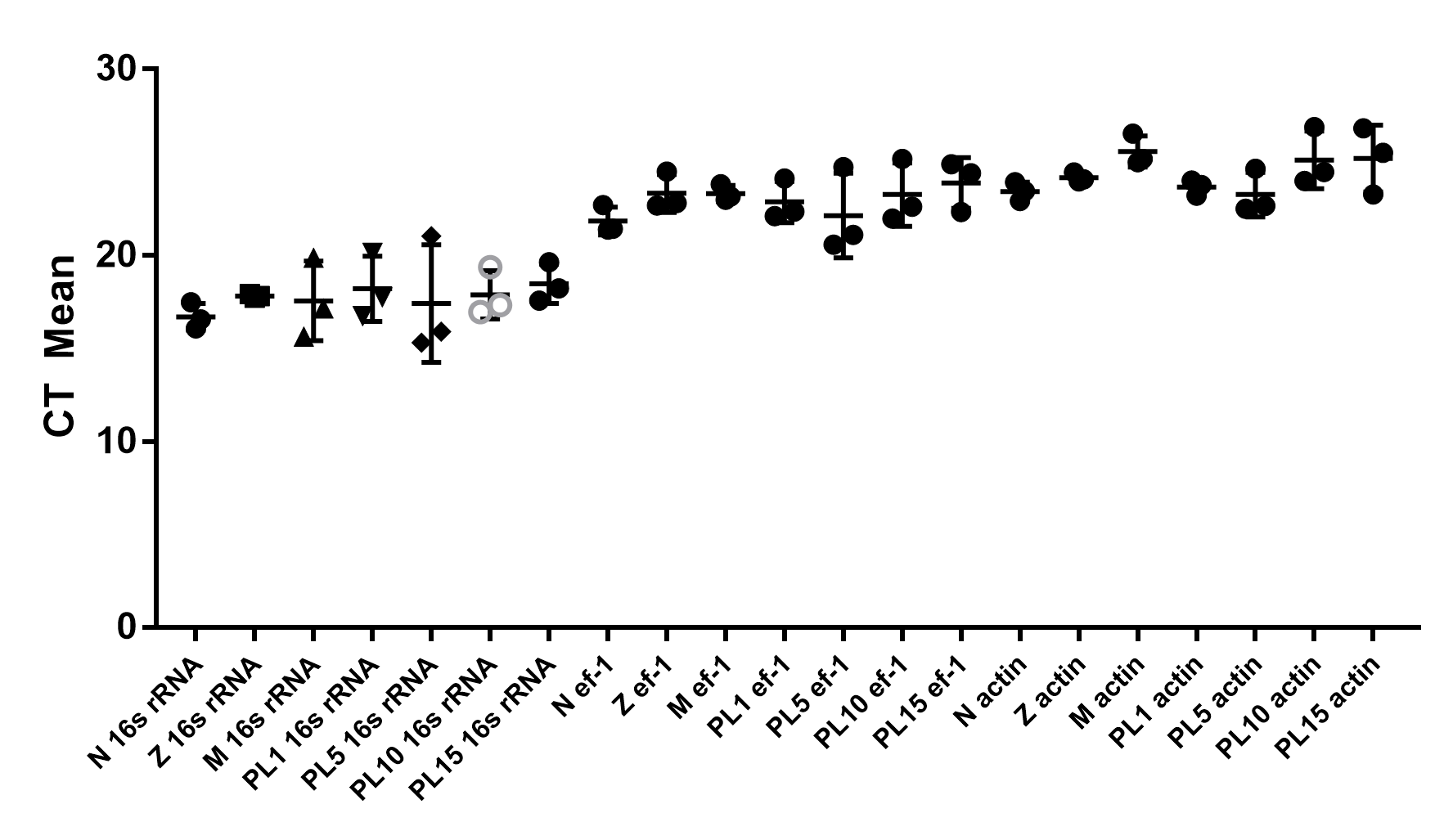

Supplement: Supplemental Information 4 [file peerj-10-12980-s004.png]

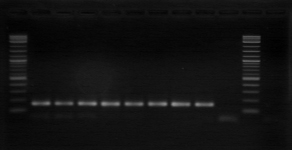

Supplement: Supplemental Information 10 — Tissue distributions of PmOvtra 2, Pmfru-1, Pmfru-2, and 16s rRNA. [file peerj-10-12980-s010.zip › 16s rRNA gel pic.jpg]

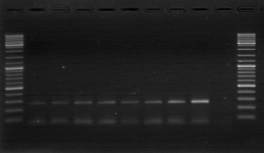

Supplement: Supplemental Information 10 — Tissue distributions of PmOvtra 2, Pmfru-1, Pmfru-2, and 16s rRNA. [file peerj-10-12980-s010.zip › fru-1 gel pic.jpg]

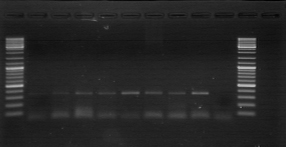

Supplement: Supplemental Information 10 — Tissue distributions of PmOvtra 2, Pmfru-1, Pmfru-2, and 16s rRNA. [file peerj-10-12980-s010.zip › fru-2 gel pic.jpg]

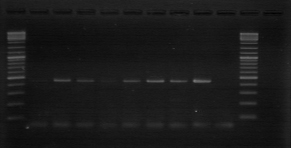

Supplement: Supplemental Information 10 — Tissue distributions of PmOvtra 2, Pmfru-1, Pmfru-2, and 16s rRNA. [file peerj-10-12980-s010.zip › PmOvtra2 gel pic.jpg]
