# Supplementary material for: Molecular characterization and expression profiling of transformer 2 and fruitless-like homologs in the black tiger shrimp, Penaeus monodon
Source: PeerJ. 2022 Feb 17;10:e12980. doi: 10.7717/peerj.12980 (PMC8858584; doi:10.7717/peerj.12980)
Supplement: Supplemental Information 6 [file peerj-10-12980-s006.docx]

**Penaeus monodon fruitless isoform 2 (Pmfru-2)**

GATCGATTGTTTACTTGTCTGTCCTGGAGTGC

CCTTTGGAAACCATTTTTAAAAGACAAATTACACTGACGCTTCGAGAAAGACATTAAGAG

ATGGAGGAGGGTATACTCTCACTGCGTTGGAATAATCATCGATCAACATTTTTCCATATT

TTATCTACACTTCATAGGAAGGAACTTTACAGTGATGTAACATTAGCATGCAATGGTAAA

TTTTTCCCAGTACACAAGTTGGTGCTTTCAGTGTGTAGTGAATATTTTGAGGAAATGTTT

AAGCAGACCACATGTAAACATCCCATAATAGTACTTAAGGATATTCTTCACGATGACCTT

GAAGCGCTTCTCAATTACATGTATGCGGGTGAGGCAAATGTAGCGCAAAATGACCTCGCC

AGATTAATTAAAGCTGCTGAGTGTTTAAGAATCAAAGGTCTAGCCGTCCCGGATGAAGCC

CCCCCTTCCAGTGAGAGTAAAAGATCCCATACTGAGGGGCTGAGAGAGGAAGCGCCTCAC

CCCAAGCGGCGGAAACATGATGACAGCTCATCAGCCTCCTCCAAGTCGAGCCAAGGGCGA

CAGTCAGAGGATGAACCCAAAGATAAAAATTGCAAGGAATTGCCTAGCTGCATGGAACAA

CAGCAACAACATAGTGGAAGATATAGTGGGCAAAGTACAGGATTACAACAAATAACGAGT

CCTGAACTACAACTTGAATTAGAAATGGGGAGATCAAGTCAAGACGACAACAGTGCTACT

CAAGATCTTGCTGAGGTTGTCCTGGATGAACAGCCGCTGATCAAAGAGGAGATCCAGGAA

CCCAAACATGAACATGATGACGACATAACGCACCAAACGGACTCGGAGGCAAGCATCAGT

TTCGACCCCCTCAATTCTGGGGATGAAAGAGGAGGGGGTACAGGCATATATGACCCACAG

CTGATGGTCTCACACCCACAGAGTGTCCTACAAGATATCATGGTGCAAGGGGTCCCGGGC

CCTTCTGGACTGCCCACGGACTCCATCACCAGCTGGGATTCAGGTGGAAATGTAGGCTTT

TCGCTAGAAGGATTCACAGGCGAGGATGCGAGGACGACCCAGGCCATGGATGTGCGAGGC

AGTTATAGGCGAAGCCAGTTTGGAAGTAGGTGTGGCAGTGGAGCGACGGGTGGCATGGCT

GGCGGCACCAGAGGGGCACAAGTTGAAGGTGGAACCCACCCATGTGTGCTGTGCCAGAAG

CGCTTCACCAGCCGCCAGGACCTGAGACGCCACGTCCGCACTCACACAGGAGAGAGGCCA

TACCAGTGTCCGCTTTGCCCTCACCGTGCGGCGCTCAAGGGCAACATCAAGAAGCACATC

ATAGCAGTCCACAGAGACATAACCCCGACTGCAGCTGAGGCTGCAGCAGTTCTTGAGAAA

GAATCATTCCAGGCAGCTGACAACAATGCTGGTGCTGCTACCACCTCCAACACTGGTGTT

GGTGTTGAGCTCTTACAGAACTGTTCTTACTCACCAGAGAGTTTCCCTAATTGTTGAAAG

ATATGTGGAAGCTTTGTAGAAGAATTTTTTTTGACAGAGAGATATTAAGTAATTTCCATA

ATTGGTTATAATCCTCATTACCATCTTGAATATTATTTATGAATTGATATATGAGACATT

ATTTTTTTTAAGTGATATATGTAATTATCCTTACTGGTAGGTCTTAGCTGTATCTATGAT

ATATTGATTTAAGTTACAGTGTAAAAAAATACCAGGCACACCTCATAACCCAAAAGTAAA

TGAAAATGCATTAGGGATATGATATACAAGGTTTGCCTGTATAACAGTTCAGTACAAGCA

CTATTACACAAGTAAACTTCCTGAAG
