# Supplementary material for: Molecular characterization and expression profiling of transformer 2 and fruitless-like homologs in the black tiger shrimp, Penaeus monodon
Source: PeerJ. 2022 Feb 17;10:e12980. doi: 10.7717/peerj.12980 (PMC8858584; doi:10.7717/peerj.12980)
Supplement: Supplemental Information 12 [file peerj-10-12980-s012.docx]

*P. monodon ovarian associated transformer 2* (*PmOvtra 2)*

AGCGAGGAGCGAGTGCGGACGGACGGACTGCGTCTCGGTTTTCCTCTCGTTTGACTGCGATTTTGCCGCGAGATTGAGTCGTTCCGGAGGCGGAGGCGAGGTACAGAAGAATGAGTCGATCACCGCAACATGCTGTGGCCAATGGAGCCTCACCAGTTCGTGAAAGGTCGAGGGACCCTTCATTTTCCCGCTCAAGATCACGCTCCAGAGACCGACGTGAAACCTACAAACACTCCTCTAGAAGTTCTGGTTCCCCACGTTACCGCGAGGACCGCTACCGAGAGGACAAATATTCATCATCACGCCGTAGAGATTCTCGTTCTCCATCTTACTCCAGAAATAGAAGGAGTAACAGGTCACCAATGTCGAATCGCCGCCGCCATCATGGAAGCAGAGAGGACCCGTCCCCGAGCAACTGCCTTGGCATCTTTGGACTCTCTCTCTACACAACCGAGAGACAGCTTCATCACCTCTTTGGCAAATATGGTCATATCAACGAAGTTCAAGTTGTACTAGATGCTAAAACTGGTCGATCAAGGGGATTTGCATTTATCTACTTTGATCATGTGGATGATGCCACAGAAGCCAAGGAGCAGTGTACTGGGATGGAGATTGATGGAAGACGGATCAGAGTAGATTATTCCATCACAGAGAGAGCCCACACACCCACTCCTGGCATATACATGGGTCGACCAACTTATTCTAACAATGGTCGTAGAGGTGGTGGTGGAGGTGGAGGAGGTCGCCACAGAGGTGGATATGGAGGAGGCAGTCGCAGTCGCCGGTCCCCTCCACGCAGATCATACCGCTCACGCTCTCGGTCATACTCTCCACGTCGCTACAGCCGGTATTGAGGTGCTGGTGTCACTTTAGTTTGATTATAACTAGAAACATGAACTGTATTTAATGTAACTACACATTCTAACAAGATCAAAGCACTCTGATATAAAAAGCATATTTTATCCTGTTAGAATGCAACATTAGATATCCAGCAGTTTCATGGTTTTCAAAGTTTAGCAGACCTATATTTAAGGACAAGCTGGTTGTTTTGACCAACAGTTTTTCTATGTACTGAGGAAGTATTCCTCAAGATATAATGTTCTTTGCATCAGGTATCAAGATATTTTTCGTTAAAAGATGACCAGAAGTGAATATCTGCTGTGTCTTGAGATATAATAAGCATTTTATATATGGAGAGGAATGAAAGAAACAAGATTATTTTCAAATTTAGGACTTTTTCATCTTATCAGGAGAATCCAGAATGAATATTTCTATTTTTTGTCTTCTGTGTATTTCTTTTCTTGTTTTAAGATGAGTTTTATTAGTATTTTGGAGTGATTCTAGGTCAATAATCACCCTTTCTGTAACACATTATAAAATGATCAATAAAGATGAAACAAAAGGGTTGTTTAATAGGCATTAGTTCTCCTTGTAGATAGTTGATTTACAATACCTTTGTATGTTGAGAACTTCTTTGATTGTTTCATCCATCTCAGTTTGTTTCGTAAAAGACATTGCTCACGAGTTTGCCACAACTTGGTATATACTGATTATGTTCTGTTATGGTTTCCCTTTTTTCTCTTTTTATCTTTTCATAAGTGAATGACAAACATGTGCTTATTCATTGGTAAGTGAAAGTAATGTGAAAGTGATTAAAGGTGACCCATGTTTAGACTAGATGGAGTTGAGGTGGGACATCCTCATTAAAGGAAAAAGAATTACTTAAGTTGAAGCTTAAGACTGTGTACGTCAGAAGTTTTTTTGTGGTTCGCTC
